# Supplementary figures and images for: CD40L Activates Platelet Integrin αIIbβ3 by Binding to the Allosteric Site (Site 2) in a KGD-Independent Manner and HIGM1 Mutations Are Clustered in the Integrin-Binding Sites of CD40L
Source: Cells. 2023 Jul 31;12(15):1977. doi: 10.3390/cells12151977 (PMC10416995; doi:10.3390/cells12151977)

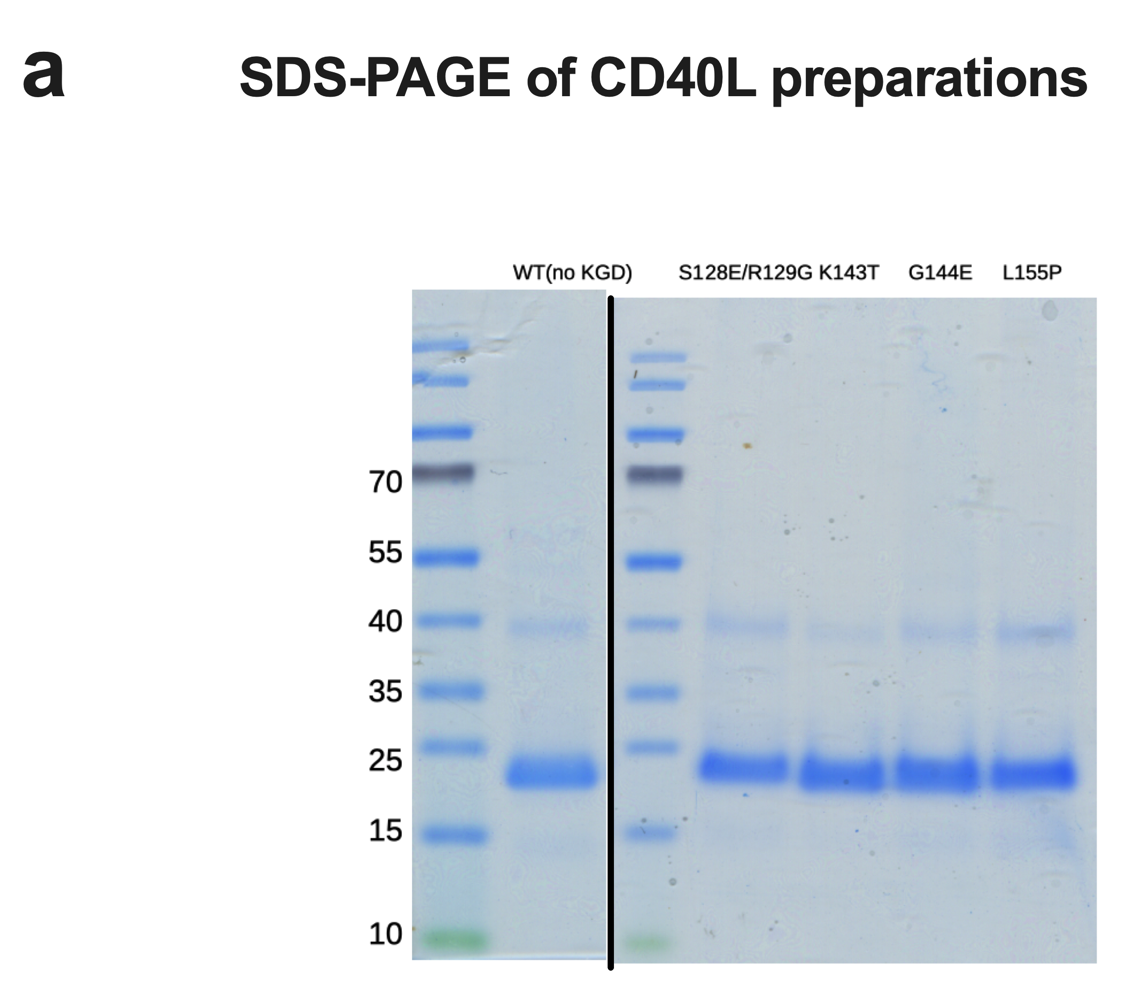

Supplement: Supplementary file 1 [file cells-12-01977-s001.zip › cells-2392879-supplementary.tiff]
